# Phylogenetic analyses of bat-associated bugs (Hemiptera: Cimicidae: Cimicinae and Cacodminae) indicate two new species close to *Cimex lectularius*

**DOI:** 10.1186/s13071-017-2376-1

**Published:** 2017-09-21

**Authors:** Sándor Hornok, Krisztina Szőke, Sándor A. Boldogh, Attila D. Sándor, Jenő Kontschán, Vuong Tan Tu, Ali Halajian, Nóra Takács, Tamás Görföl, Péter Estók

**Affiliations:** 1 Department of Parasitology and Zoology, University of Veterinary Medicine, Budapest, Hungary; 2 Department of Nature Conservation, Aggtelek National Park Directorate, Jósvafő, Hungary; 3 Department of Parasitology and Parasitic Diseases, University of Agricultural Sciences and Veterinary Medicine, Cluj-Napoca, Romania; 4 Plant Protection Institute, Centre for Agricultural Research, Hungarian Academy of Sciences, Budapest, Hungary; 5 Institute of Ecology and Biological Resources, Vietnam Academy of Science and Technology, Hanoi, Vietnam; 6 Department of Biodiversity, School of Molecular and Life Sciences, University of Limpopo, Sovenga, South Africa; 7 Department of Zoology, Hungarian Natural History Museum, Budapest, Hungary; 8 Department of Zoology, Eszterházy Károly University, Eger, Hungary

**Keywords:** Cytochrome *c* oxidase subunit 1, *cox*1, Internal transcribed spacer 2, ITS2, *Cacodmus*, *Cimex pipistrelli*

## Abstract

**Background:**

Bats are regarded as the primary (ancestral) hosts of bugs of the family Cimicidae. The historically and economically most important species in the family is the common bedbug (*Cimex lectularius*), because of its worldwide occurrence and association with humans. This molecular-phylogenetic study was initiated in order to expand the knowledge on the phylogeny of cimicid bugs of bats, by investigating samples from Hungary, Romania (representing central-eastern Europe) and two further countries (South Africa and Vietnam).

**Results:**

Altogether 216 cimicid bugs were collected (73 *Ci. lectularius*, 133 *Ci. pipistrelli*, nine *Cacodmus ignotus* and one *Ca. sparsilis*). Members of the *Cimex lectularius* species group were found both in the environment of bats (only *Myotis emarginatus*, which is a cave/attic-dwelling species) and on three crevice-dwelling bat species (two pipistrelloid bats and *M. bechsteinii*). On the other hand, *Ci. pipistrelli* always occurred off-host (near *M. myotis/blythii*, which are cave/attic-dwelling species). In addition, two *Cacodmus* spp. were collected from *Pipistrellus hesperidus*. The morphological characters of these specimens are illustrated with high resolution pictures. Analysis of cytochrome *c* oxidase subunit 1 (*cox*1) sequences generated from 38 samples indicated relative genetic homogeneity of *Ci. pipistrelli*, while the *Ci. lectularius* group had two haplotypes (collected from pipistrelloid bats in Hungary and Vietnam) highly divergent from other members of this species group. These results were confirmed with molecular and phylogenetic analyses based on the internal transcribed spacer 2 (ITS2). Bat-associated bugs morphologically identified as *Ca. ignotus* and *Ca. sparsilis* were different in their *cox*1, but identical in their ITS2 sequences.

**Conclusions:**

Molecular evidence is provided here on the existence of two new genotypes, most likely new species, within the *Ci. lectularius* species group. The relevant specimens (unlike the others) were collected from pipistrelloid bats, therefore the association of *Ci. lectularius* with different bat host species (pipistrelloid *vs* myotine bats) should be evaluated further as a possible background factor of this genetic divergence. In addition, *Ca. ignotus* is reported for the first time in South Africa.

## Background

Cimicid bugs (Hemiptera: Cimicidae) include approximately 110 described species [1], which are obligate, haematophagous ectoparasites of birds and mammals. Bats are regarded as the primary (ancestral) hosts of bugs in the Cimicidae, with subsequent switches to other hosts, including birds and humans [2–4]. This is well reflected by the fact that the majority (approx. two-thirds) of cimicid species are bat-associated [2, 5].

The historically and economically most important species in the family is the common bedbug (*Cimex lectularius*), because of its worldwide occurrence and preference of human environment. While the number of reported cases of bedbug infestations had been showing a decline until the middle of twentieth century, during the last two decades *Ci. lectularius* became an emerging pest even in highly developed countries [6]. This is the most important aspect that may illustrate the growing significance of this species, but not the only one. *Cimex lectularius* is the potential vector of at least 65 pathogens [6], but its vector competency awaits verification.

The morphology of cimicid bugs is frequently a matter of controversy. For instance, in the *Ci. pipistrelli* group, the morphological characters intended to delineate species were shown to vary significantly enough to ascribe progeny of the same female to different species [5, 7]. Accordingly, the taxonomy of bat-associated bugs is currently in a state of transition. While some formerly distinguished *Cimex* species are suggested to be synonymous (as exemplified by *Ci. pipistrelli* and *Ci. dissimilis*, see [5]), new species are also discovered/described [8]. The genus *Oeciacus* (associated with birds) has been transferred to *Cimex* [4]. These and other examples highlight the importance of molecular phylogenetic studies focusing on cimicid bugs, which recently have started to expand [5, 9].

Bat-associated bugs, as well as other cimicid bugs, are temporary ectoparasites, which spend most of their life off-host. On the other hand, bat-associated bugs are unable to fly, therefore strictly rely on their hosts for colonization of new habitats, as well as for distribution over large distances [5, 10]. Cimicid bug species associated with bats show different host ranges [2, 11]. Adaptation to host species has been suggested to be a driver of morphological rather than genetic diversification in the case of *Ci. pipistrelli* [5]. At the same time, host preference will also influence the small-scale (habitat-related) as well as large-scale (geographical) distribution of bat-associated bugs [9, 11, 12].

While *Ci. lectularius*, as a man-associated parasite, has a worldwide geographical distribution [6, 9], accounts of its bat-related lineages concentrate in the Western Palaearctic [9, 11]. In this region a recent phylogeographic study on *Ci. lectularius* revealed that this species is currently undergoing lineage divergence through host association [9]. However, in the latter survey Hungary was underrepresented, and in another comprehensive survey on bat-associated bugs [5] the Balkans and other regions of the Old World had not been included. This molecular-phylogenetic study was initiated in order to expand the knowledge on the phylogeny of cimicid bugs of bats, by investigating samples from Hungary, Romania (the latter representing the Balkans) and two further countries (South Africa and Vietnam).

## Methods

### Sample collection and study design

Bat-associated cimicid bugs were collected in Hungary (six locations), Romania (two locations), South Africa (one location) and Vietnam (one location) between 2011 and 2016 (Table 1). The bats were caught for monitoring and ringing purposes at cave entrances from sunset to dawn, using harp traps or Ecotone mist-nets (Gdynia, Poland) with standard 12 m length, 2.5 m height and 14 × 14 mm mesh size. Bats were released immediately after parasite removal and recording data (date and place of collection, bat species). Alternatively, the close environment of nursing bat colonies with different habitat types (Table 1) was checked for the presence of Cimicidae. The bugs were immediately placed into and stored in 96% ethanol.

**Table 1:** Data for the samples used in this study

Bug species or species group	Country	Location (no. of samplings × habitat type)	Host nearby (^a^on host)	Stage			GenBank ID (no. of samples amplified)	
				Nymph	Female	Male	*cox*1	ITS2
*Cimex lectularius* group	Hungary	Dráva (1 × A)	*Myotis emarginatus*	1	3	4	MF161525 (2×)	ns
		Trizs (3 × CH)		13	19	23	MF161526 (2×); MF161527 (6×)	MF161534^b^ (2×); MF161535 (1×)
		Ragály (1 × LH)		1	4	1	MF161522 (3×)	MF161535 (1×)
		Nagyvisnyó (1)	*Pipistrellus pipistrellus* ^a^	0	1	0	MF161521 (1×)	MF161532 (1×)
		Noszvaj (1)	*Myotis bechsteinii* ^a^	0	0	1	MF161520^b^ (1×)	ns
	Vietnam	Thanh Hoa, Ngoc Khe (1)	*Hypsugo pulveratus* ^a^	0	1	1	MF415647 (1×)	MF161540 (1×)
*Cimex pipistrelli*	Hungary	Szőlősardó (2 × CH)	*Myotis myotis/blythii*	3	2	4	MF161523^b^ (5×)	MF161533^b^ (4×)
	Romania	Sant (1 × M)	*Myotis myotis*	1	0	0	nd	nd
		Leghia (6 × M)	*Myotis blythii*	32	49	42	MF161524 (5×); MF161528 (2×)	MF161536 (2×); MF161537 (1×)
*Cacodmus ignotus*	South Africa	Makhado (2)	*Pipistrellus hesperidus* ^a^	0	2	7	MF161529^b^ (8×); MF161530 (1×)	MF161538^b^ (2×)
*Cacodmus sparsilis*				0	0	1	MF161531 (1×)	MF161539 (1×)

*Abbreviations*: *A* attic, *CH* church tower, *LH* lich house, *M* mine, *ns* not successful, *nd* not done

^a^On host

^b^Used as reference sequence of the given bug species in the text

Concerning study design, because the size of structures important for morphological identification of cimicid bugs was shown to exhibit significant intraspecific variation [2, 5], detailed measurements were not taken, and only discrete morphological characters were considered for species identification.

Morphological identification of adult bugs was carried out under a stereomicroscope (SMZ-2 T, Nikon Instruments, Japan) by using standard keys [2], focusing on the pronotum, paragenital sinus (*Cimex* spp.) or the paramere (*Cacodmus* spp.). Concerning samples (one from Hungary and two from Vietnam), which showed high degrees of genetic divergences from other members of their phylogenetic group, their conspecificity with *Ci. emarginatus* (reported in Bulgaria) and *Ci. insuetus* (reported in Thailand), respectively, was excluded based on descriptions of the latter species [8, 13]. *Cacodmus* sp. females were identified according to the *cox*1 sequences of morphologically identified males. Pictures were made with a VHX-5000 (Keyence Co., Osaka, Japan) digital microscope.

### DNA extraction and molecular analyses

DNA was extracted from individual bugs with the QIAamp DNA Mini Kit (Qiagen, Hilden, Germany) according to the manufacturer’s instruction, including an overnight digestion in tissue lysis buffer and Proteinase-K at 56 °C. Molecular phylogenetic analysis was attempted from 44 samples, including 1–10 adult bugs from each location.

The cytochrome *c* oxidase subunit 1 (*cox*1) gene was chosen as the primary target for molecular analysis, on account of its suitability as a DNA-barcode sequence for cimicid bug species [3]. The PCR amplifies a 658 bp long fragment of the *cox*1 gene of various insect orders. The primers Lep1F (5′-ATT CAA CCA ATC ATA AAG ATA TTG G-3′), Lep1Fdeg (5′-ATT CAA CCA ATC ATA AAG ATA TNG G-3′) and Lep3R (5′-TAT ACT TCA GGG TGT CCG AAA AAT CA-3′) [4] were used in a reaction volume of 25 μl, containing 1 U (0.2 μl) HotStarTaq Plus DNA polymerase, 2.5 μl 10× CoralLoad Reaction buffer (including 15 mM MgCl_2_), 0.5 μl PCR nucleotide Mix (0.2 mM each), 0.25 μl (0.5 μM final concentration) of each Lep1F and Lep1Fdeg primers and 0.5 μl (1 μM final concentration) of Lep3R primer, 15.8 μl ddH_2_O and 5 μl template DNA. For amplification, an initial denaturation step at 95 °C for 5 min was followed by 40 cycles of denaturation at 94 °C for 40 s, annealing at 53 °C for 1 min and extension at 72 °C for 1 min. Final extension was performed at 72 °C for 10 min. In addition, a similar length fragment of the *cox*1 gene of the sample from Vietnam was amplified with the primers HCO2198 (5′-TAA ACT TCA GGG TGA CCA AAA AAT CA-3′) and LCO1490 (5′-GGT CAA CAA ATC ATA AAG ATA TTG G-3′) [14] as reported [15].

To complement the results obtained with the mitochondrial *cox*1 gene, 16 samples that showed different *cox*1 haplotype within a country, were also tested for a nuclear marker, the internal transcribed spacer 2 (ITS2). This PCR amplifies a ~1027 bp fragment of the ITS2 of Hemiptera [16], with the primers CAS5p8sFc (5′-GCG AAC ATC GAC AAG TCG AAC GCA CAT-3′) and CAS28sB1d (5′-TTG TTT TCC TCC GCT TAT TAA TAT GCT TAA-3′). Five μl of template DNA were added to 20 μl reaction mixture, containing 1 U (0.2 μl) HotStarTaq Plus DNA polymerase, 2.5 μl 10× CoralLoad Reaction buffer (including 15 mM MgCl_2_), 0.5 μl PCR nucleotide Mix (0.2 mM each), 0.5 μl (1 μM final concentration) of each primers and 15.8 μl ddH_2_O. An initial denaturation step at 95 °C for 5 min was followed by 35 cycles of denaturation at 95 °C for 30 s, annealing at 59 °C for 40 s and extension at 72 °C for 1 min. Final extension was performed at 72 °C for 7 min.

PCR products were visualized in 1.5% agarose gel. Purification and sequencing were done by Biomi Inc. (Gödöllő, Hungary). Obtained sequences were manually edited, then aligned and compared to reference GenBank sequences by nucleotide BLASTN program (https://blast.ncbi.nlm.nih.gov). Representative sequences (including identical haplotypes from different locations) were submitted to GenBank (accession numbers: MF161520–MF161531 for *cox*1, and MF161532–MF161540 for ITS2). Phylogenetic analyses were conducted by using MEGA version 6.0, with the Maximum Likelihood method and the model (Tamura 3) selected by the program.

## Results

### General morphology and host species of bat-associated bugs

Altogether 216 cimicid bugs were collected from the bodies or roosts of seven bat species of three genera (Table 1). Bugs morphologically most closely related to *Cimex lectularius* were found both in the environment of bats and on the bat species *Pipistrellus pipistrellus*, *Myotis bechsteinii* and *Hypsugo pulveratus*. On the other hand, *Ci. pipistrelli* occurred only off-host (Table 1).

**Fig. 1:**
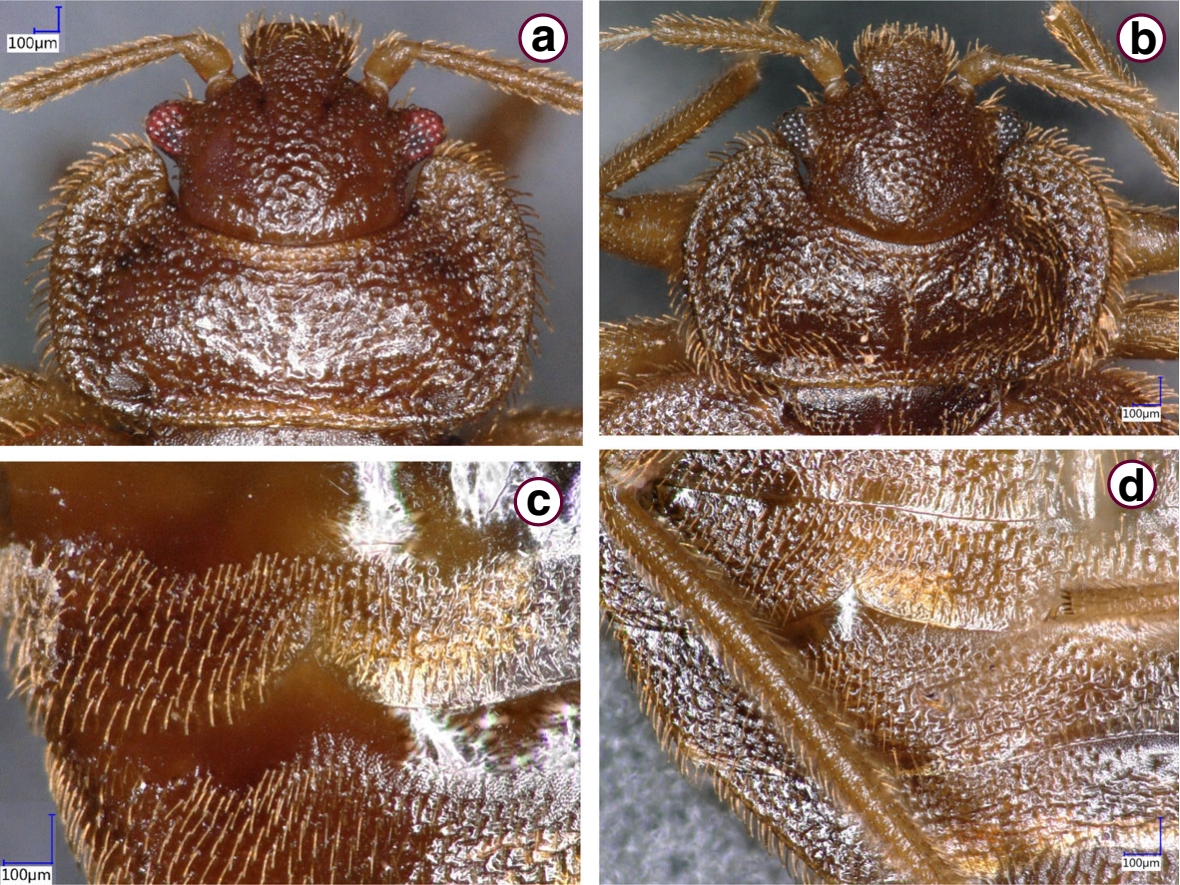
*Cimex lectularius*, females collected near *Myotis emarginatus*, in three locations of Hungary: Dráva (**a**, **c**); Trizs (**b**); and Ragály (**d**). **a**, **b** Head and pronotum. **c**, **d** Paragenital sinus

**Fig. 2:**
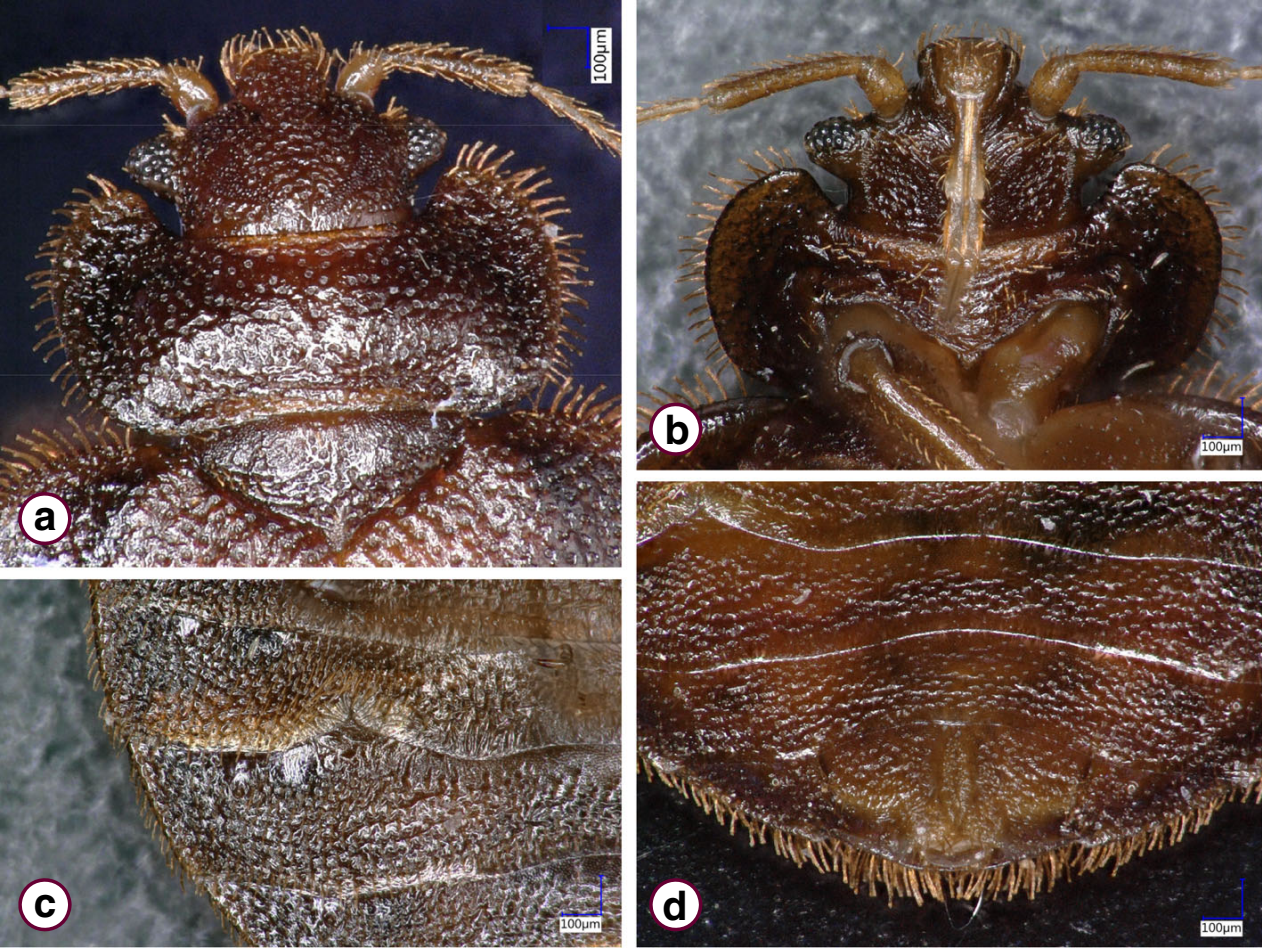
*Cimex* sp., female collected from *Pipistrellus pipistrellus* in Hungary (Nagyvisnyó). **a** Head and pronotum, dorsal view. **b** Head and pronotum, ventral view. **c** Paragenital sinus. **d** Last two abdominal segments

**Fig. 3:**
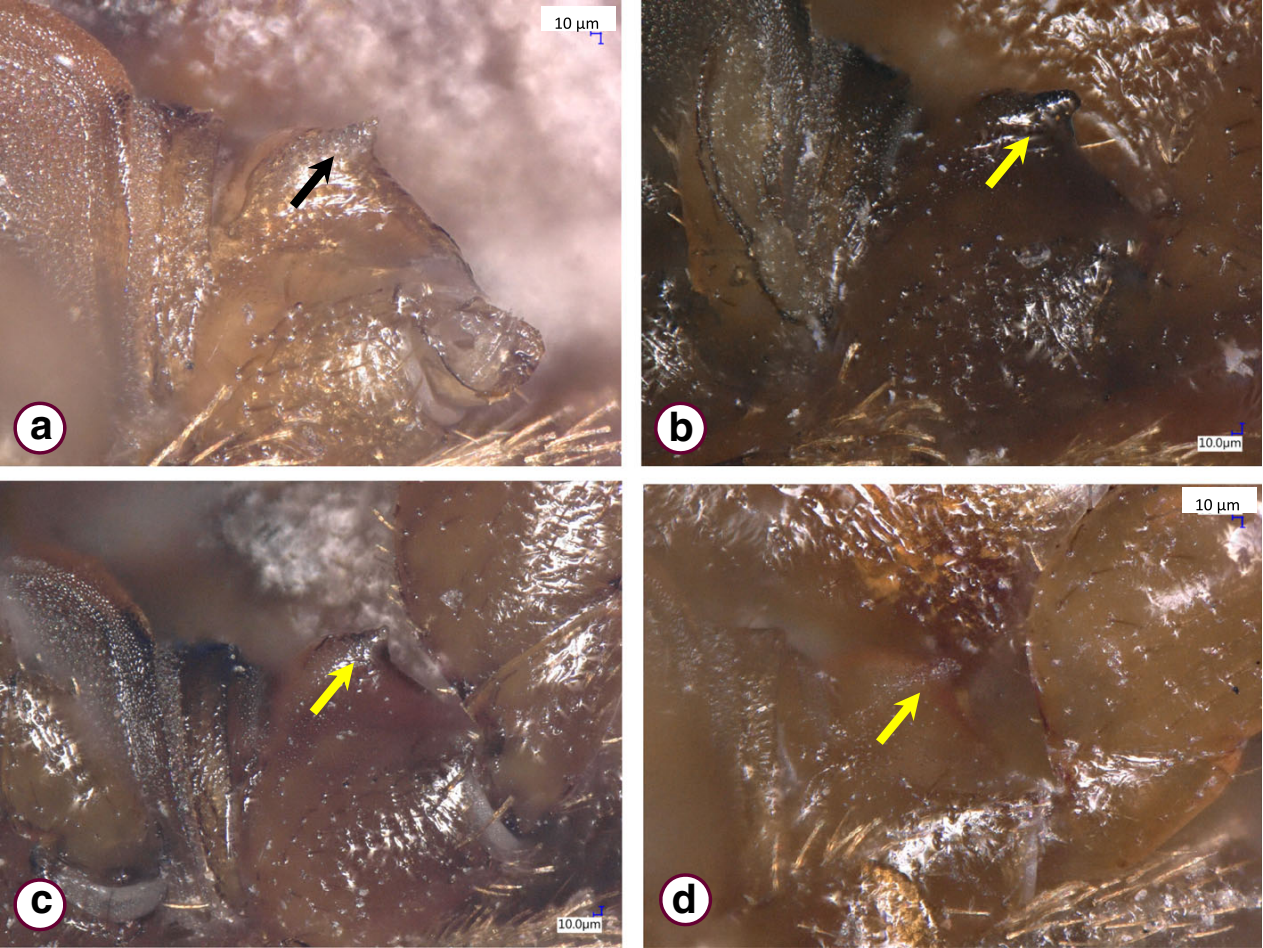
*Cimex lectularius*, broad basis (arrow) of external spur on coxa III. Specimens collected in Hungary from *Pipistrellus pipistrellus* (Nagyvisnyó) (**a**) and *Myotis bechsteinii* (Noszvaj) (**b**); in a human dwelling (Budapest, Neptun street) (**c**); and a specimen collected in Vietnam from *Hypsugo pulveratus* (**d**)

**Fig. 4:**
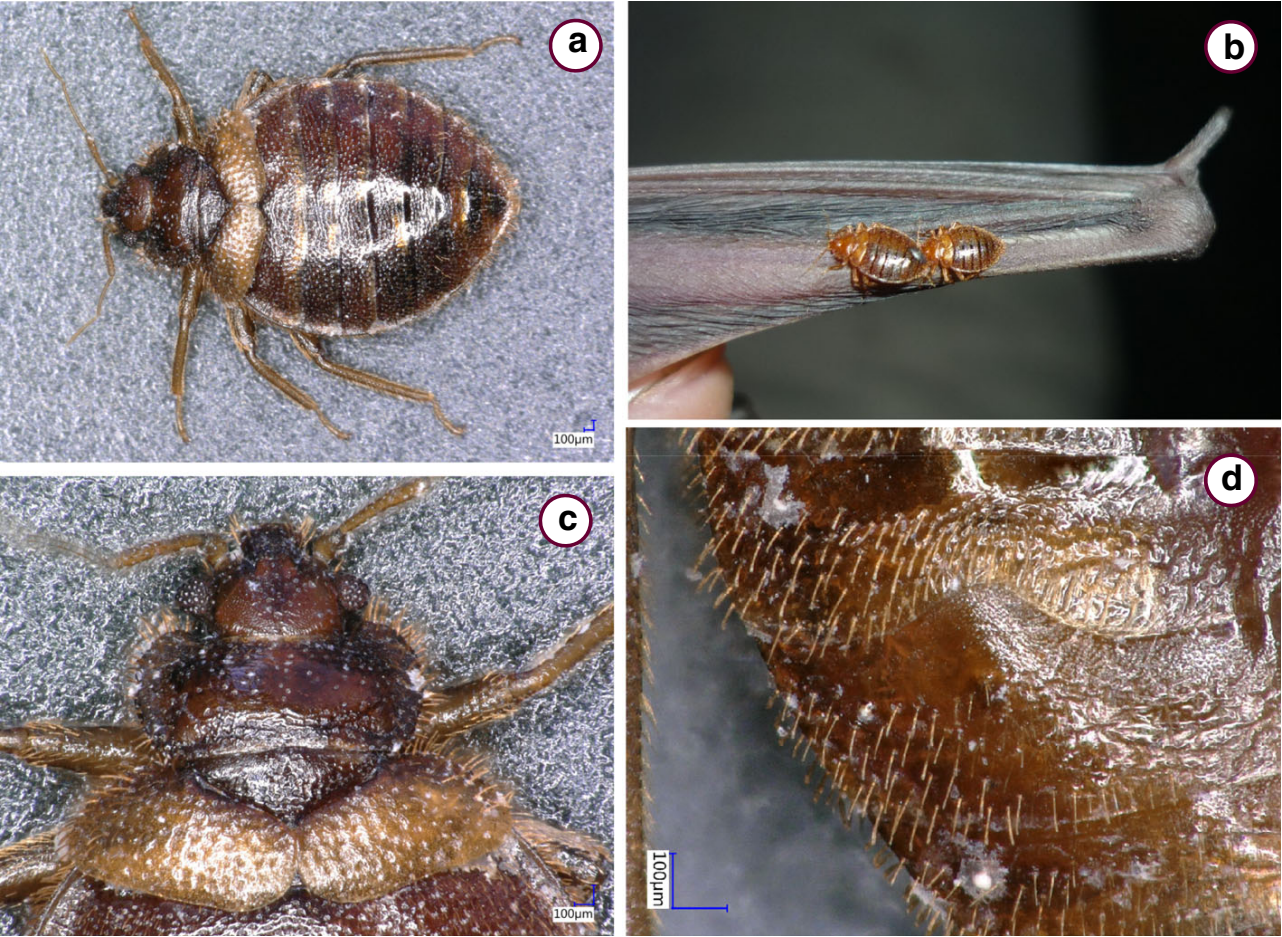
*Cimex* sp., female collected from *Hypsugo pulveratus* in Vietnam. **a** Habitus. **b** In situ on bat patagium. **c** Head and pronotum. **d** Paragenital sinus

The *Cimex lectularius* species group was represented by 73 specimens. These showed similar general morphological characters if collected near bats (Fig. 1) or from bats in Hungary (Figs. 2 and 3b), including the shape of the pronotum (breadth to length ratio ≥ 2.5, broad lateral lobes), paragenital sinus (cleft with bristles) and external spur on coxa III (with a broad basis). However, while the pronotum and coxal spur were similar in the case of specimens from Hungary (Fig. 3a-c) and Vietnam (Fig. 3d), the paragenital sinus of the female bug from Vietnam was rounded (Fig. 4d).

**Fig. 5:**
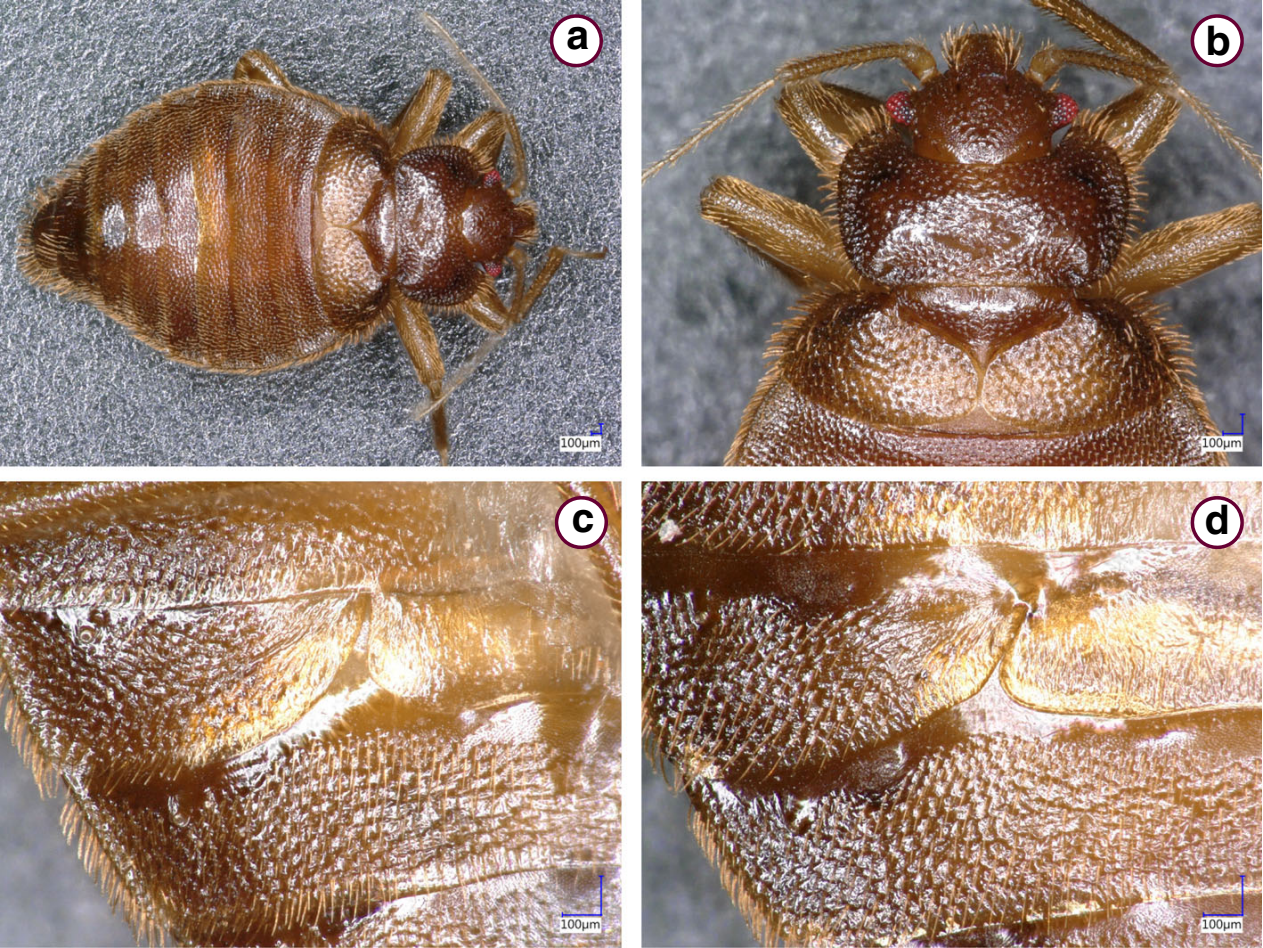
*Cimex pipistrelli*, female collected near *Myotis* spp. **a**-**c** Specimen collected in Hungary (Szőlősardó). **a** Habitus. **b** Head and pronotum. **c** Paragenital sinus. **d** Specimen collected in Romania (Leghia), paragenital sinus

*Cimex pipistrelli* was represented by 133 specimens (Table 1). All of these from Hungary and Romania shared the shape of the pronotum (breadth to length ratio < 2.5, narrow lateral lobes) and of the paragenital sinus (cleft and naked) (Fig. 5).

**Fig. 6:**
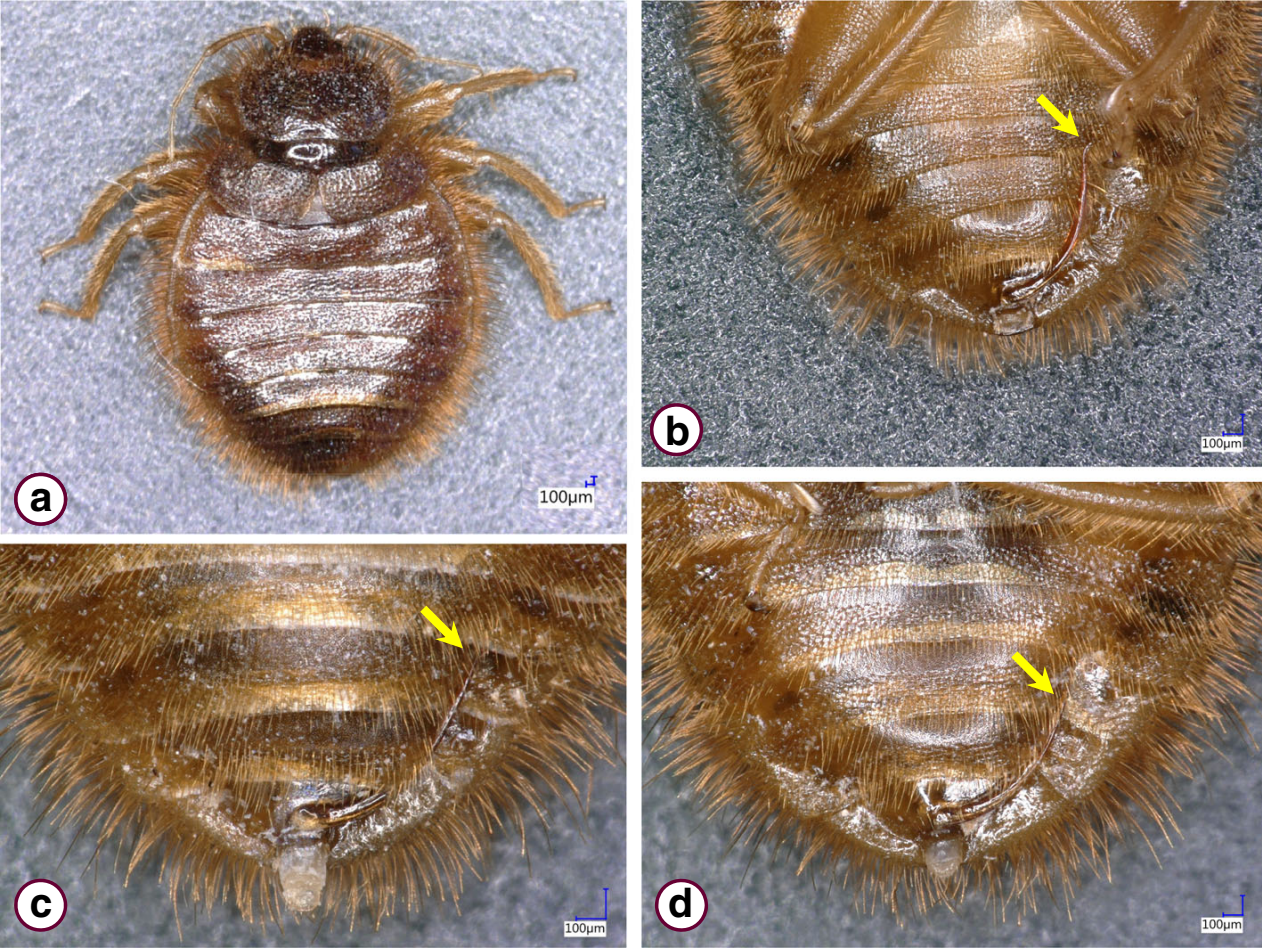
*Cacodmus* spp. collected from *Pipistrellus hesperidus* in South Africa (Makhado). **a** *Ca. ignotus*, habitus. **b** *Ca. sparsilis* male with long (> 1000 μm) paramere, curved apically. **c** *Ca. ignotus* male with medium length paramere (850 μm). **d** *Ca. ignotus* male with slightly longer paramere (950 μm). Arrows indicate paramere apex

*Cacodmus* sp. males from South Africa had either evenly curved and tapering, apically straight, medium to long paramere (Fig. 6c-d), or long paramere bent laterally at the tip (almost sinuate at apex) (Fig. 6b). On this basis specimens were assigned to *Ca. ignotus* and *Ca. sparsilis*, respectively.

### Sequence comparison and phylogeny of bat-associated bugs

The *cox*1 gene fragment was successfully amplified and sequenced from 38 samples (Table 1). Bugs morphologically most closely related to *Ci. lectularius* (15 samples) had four *cox*1 haplotypes in Hungary. The majority of these exhibited up to five nucleotide differences from each other, corresponding to 99.2–100% sequence similarity (626–631/631 bp). However, a *Cimex* sp. from *P. pipistrellus* (Hungary) showed 46 nucleotide differences from the *Ci. lectularius* reference sequence (MF161520: from Hungary), i.e. only 585/631 bp (92.7%) sequence similarity. The *cox*1 gene fragment of another *Cimex* sp. from Vietnam revealed an even lower, 522/631 bp (82.7%) sequence similarity with *Ci. lectularius*.

Bugs identified as *Ci. pipistrelli* (represented by 12 samples) had three *cox*1 haplotypes. These exhibited up to six nucleotide differences from each other, amounting to 99–100% sequence similarity (625–631/631 bp). *Cacodmus ignotus* from South Africa had two *cox*1 haplotypes, with only one nucleotide difference (630–631/631 bp, i.e. 99.8–100% similarity). The bug identified as *Ca. sparsilis* showed 43 nucleotide divergence from *Ca. ignotus* (588/631 bp, i.e. 93.2% similarity).

The ITS2 fragment was successfully amplified and sequenced from 16 samples (Table 1). In general, this nuclear marker showed a much lower degree of intraspecific divergence compared to *cox*1. Members of the *Cimex lectularius* group from Hungary had only two different ITS2 haplotypes. However, the *Cimex* sp. from *P. pipistrellus* (Hungary) showed only 96.7% (622/643 bp) sequence similarity in its longest region of continuous alignment with the ITS2 reference sequence (MF161534: from Hungary). In addition, the *Cimex* sp. from Vietnam showed even lower, 88.3% (580/657 bp) sequence similarity in its longest region of alignment with the ITS2 reference sequence.

*Cimex pipistrelli* had two nearly identical ITS2 sequences (941–942/942 bp, i.e. 99.9–100% similarity). Unexpectedly, samples identified as *Ca. ignotus* and *Ca. sparsilis*, which showed only 93.2% *cox*1 sequence similarity, were identical in their ITS2.

**Fig. 7:**
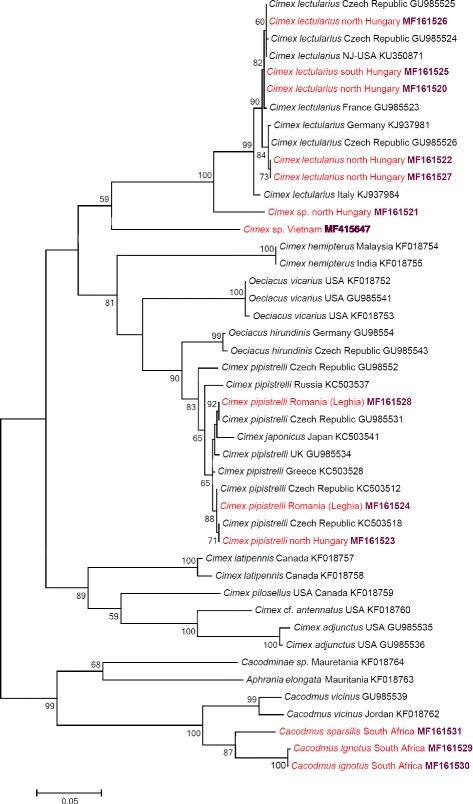
Phylogenetic tree based on the *cox*1 gene including sequences obtained in this study (indicated in red and with GenBank accession numbers in bold) and representative sequences from GenBank. Species identification is provided as in the GenBank database, although *Oeciacus* spp. were recently transferred into the genus *Cimex* [4]. Branch lengths represent the number of substitutions per site inferred according to the scale shown

**Fig. 8:**
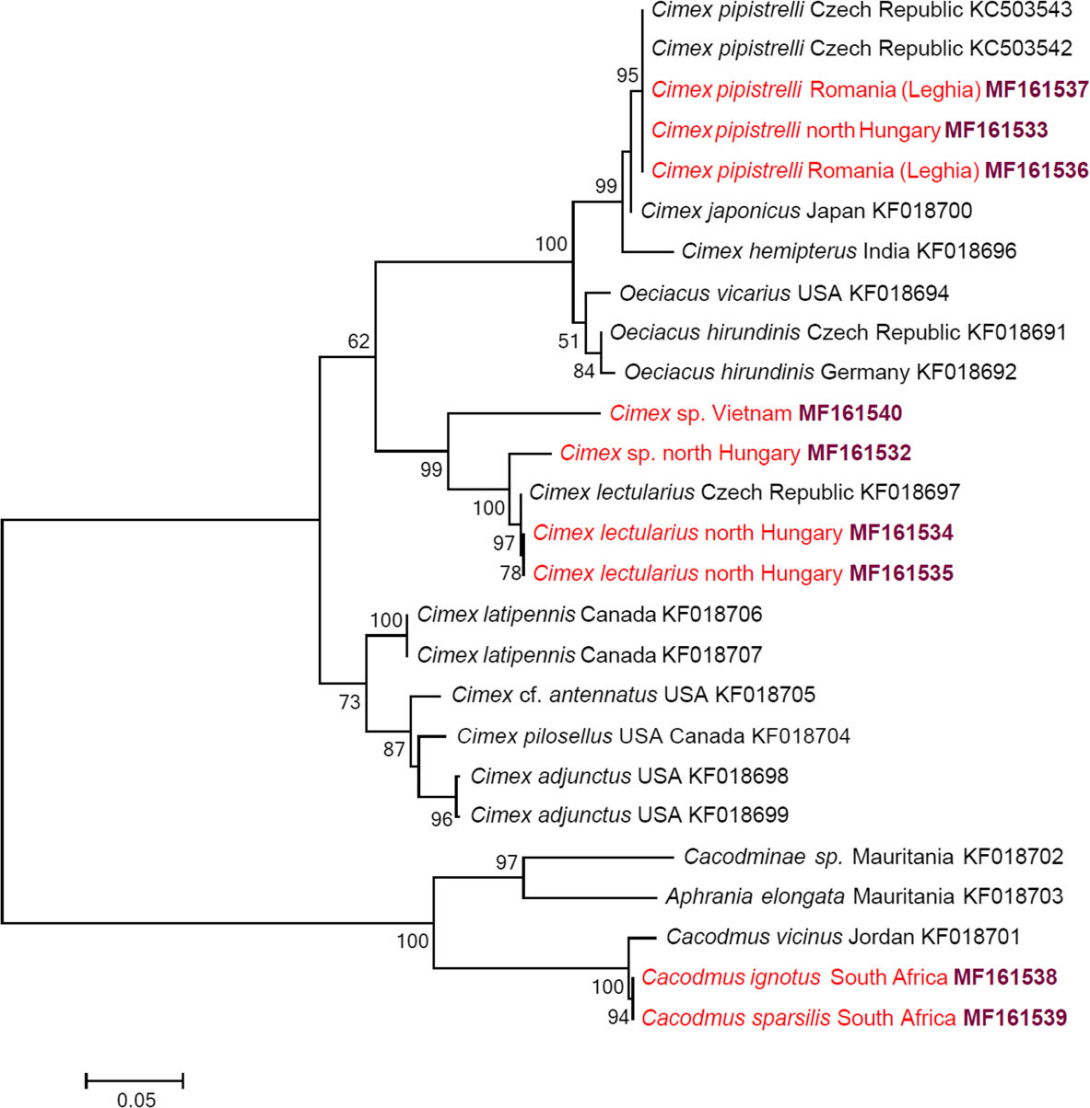
Phylogenetic tree based on the ITS2 including sequences obtained in this study (indicated in red and with GenBank accession numbers in bold) and representative sequences from GenBank. Species identification is provided as in the GenBank database, although *Oeciacus* spp. were recently transferred into the genus *Cimex* [4]. Branch lengths represent the number of substitutions per site inferred according to the scale shown

The phylogenetic relationships of *cox*1 and ITS2 sequences are shown in Figs. 7 and 8, respectively. The separation of the *Cimex* specimen (collected from *Pi. pipistrellus* in Hungary) from other isolates of the *Ci. lectularius* group was highly supported (with 100%) in both the *cox*1 and ITS2 phylogenetic analyses (Figs. 7 and 8). Similarly, the within-group separation of *Cimex* sp. from Vietnam received high (99%) support based on its ITS2 haplotype (Fig. 8), but only low (59%) support based on its *cox*1 haplotype (Fig. 7).

All *Ci. pipistrelli cox*1 haplotypes belonged to the same group with other conspecific isolates (Fig. 7), and this was confirmed in the phylogenetic analysis based on ITS2 sequences (Fig. 8). Bugs identified morphologically as *Ca. ignotus* and *Ca. sparsilis* were well separated from each other (with moderate, 87% bootstrap value) in the *cox*1 phylogenetic tree (Fig. 7).

## Discussion

This study provides molecular data of bat-associated cimicid bug species from three distant regions of the Old World (i.e. central-eastern Europe, south-eastern Asia and South Africa). One of the studied bug species, *Ci. lectularius* is the most significant member of Cimicidae, taking into account its association with humans, global distribution, historical and economic impact, recently witnessed emerging character and potential health hazards [6]. In a recent study on *Ci. lectularius* [9] mostly central and western Europe were represented by sampling sites, therefore results shown here can be regarded as complementary to that study, introducing samples from more locations in Hungary, as well as samples from Romania and Vietnam into the phylogenetic analysis of this species group. In addition, molecular analyses of *Cacodmus* spp. from South Africa have not yet been reported.

In this study all *Ci. pipistrelli*, and the majority of *Ci. lectularius* were collected in roosting places of *Myotis* spp., which can be regarded as their principal hosts [11]. Only one *Ci. lectularius* (from *M. bechsteinii*), the *Cimex* spp. (from Hungary, Vietnam) and *Cacodmus* spp. were found on hosts, in particular on four bat species, three of which are pipistrelloid bats (including *Hypsugo* [formerly *Pipistrellus*] *pulveratus*). According to literature data, bat species (such as *Pipistrellus* and *Nyctalus* spp.), which roost in narrow spaces (rock crevices or tree holes) and switch these places quite often, are more likely to carry bat-associated bugs on their wing membrane [12]. This is confirmed by the data presented here, taking into account the roosting behavior of the four bat species, which were found bug-infested (Table 1). In addition to *P. pipistrellus*, *P. hesperidus* colonies can also be found in narrow cracks and dead trees [17]. *Myotis bechsteinii* is a tree-dwelling bat species; its females establish their maternity colonies in tree holes and switch their day-roosts regularly [18, 19].

In the present study two new genotypes (belonging to the *Ci. lectularius* group, but highly divergent from its other members) were identified. Both of the relevant specimens were collected from pipistrelloid bat hosts. The first of these specimens, collected from *P. pipistrellus* in Hungary, showed the morphology of *Ci. lectularius* and was different from *Ci. emarginatus* (e.g. in the parameters of its head, palpal segments, posterior bristles and the shape of paragenital sinus). The second specimen, collected from *H. pulveratus* in Vietnam, was also similar to *Ci. lectularius* based on its coxal spur and some aspects of its pronotum (which was 2.5 times broader than long, unlike that of *Ci. insuetus*). However, the paragenital sinus of the latter female was different from that in *Ci. lectularius*, i.e. it was rounded, which is a character of Neotropical species of the genus *Cimex*, not found in the Old World [2]. In addition, taking into account that in case of both of these new variants the *cox*1 genetic difference exceeded 7% in comparison with *Ci. lectularius* (and this value was 5.8–6.4% between *Ci. lectularius* and members of the *Ci. hemipterus* or *Ci. pipistrelli* species groups, see [4]), they probably represent new species. In order to clarify the precise taxonomical status of these new genotypes, they will have to be compared by including more specimens and analyses.

While *Ci. lectularius* from roosts of *Myotis* spp. yielded multiple haplotypes within the same major haplogroup [9], in the present study *Ci. lectularius*-related specimens from pipistrelloid bats showed highly divergent *cox*1 and ITS2 haplotypes at both small and large geographical scales (i.e. in Hungary and Vietnam, respectively). This phenomenon is similar to the one suggested in the case of bat-associated bugs of the *Ci. pipistrelli* group, which were also shown to have different host ranges [2], although the association of *Ci. pipistrelli* with different host species is thought to be a driver of morphological (rather than genetic) variability [5].

The present results extend the geographical range of *Ca. ignotus* (hitherto only reported from Uganda, see [2]) to South Africa. This can be explained by the occurrence of *P. hesperidus* (from which it was collected in the present study) in much of East Africa, from Ethiopia to South Africa [20]. However, it was unexpected to find that bat-associated bugs identified here on a morphological basis as *Ca. ignotus* and *Ca. sparsilis* had highly (6.8%) different *cox*1, but identical ITS2 sequences. This observation is similar to that reported previously in the Western Palaearctic region on *Ci. pipistrelli*, which had only limited variability in ITS2 sequences (and none in other nuclear markers), despite the separation of corresponding *cox*1 haplogroups [5]. In general, the resolution of *cox*1 analysis to assess the degree of divergence between closely related species is known to be much higher compared to ITS2 (ticks: 6.1 *vs* 2.3%; mites: 3.0–4.0% *vs* < 0.5%, respectively) [21, 22]. Nevertheless, in the present case, the identity of ITS2 sequences between individuals of two *Cacodmus* spp. could have resulted from genetic introgression or hybridization.

## Conclusions

Bugs of the *Ci. lectularius* group associated with different bat host species (myotines *vs* pipistrelloid bats) were found to belong to different genetic lineages. Sequence comparisons and phylogenetic analyses of *cox*1 and ITS2 sequences of specimens from pipistrelloid bats (collected in Hungary and Vietnam) suggest that they may belong to new species. In addition, *Ca. ignotus* is reported for the first time in South Africa.

## Abbreviations

*cox*1: Cytochrome *c* oxidase subunit 1
ITS2: Internal transcribed spacer 2
